# Interleukin-6 is required for cell cycle arrest and activation of DNA repair enzymes after partial hepatectomy in mice

**DOI:** 10.1186/2045-3701-4-6

**Published:** 2014-02-03

**Authors:** Shingo Tachibana, Xiuying Zhang, Kazushige Ito, Yoshihiro Ota, Andrew M Cameron, George Melville Williams, Zhaoli Sun

**Affiliations:** 1 Department of Surgery, Johns Hopkins University School of Medicine, 720 Rutland Ave., Ross 771, Baltimore, MD 21205, USA; 2 Department of Surgery, Tokyo Medical University, Shinjuku, Tokyo, Japan; 3 School of Life Science, Tianjin University, Tianjin, China

**Keywords:** IL-6, Cell cycle arrest, Oxidative DNA damage and repair, Partial hepatectomy, Liver regeneration, Mice

## Abstract

**Background:**

Interleukin-6 (IL-6) has been shown to be vital for liver regeneration, however the specific mechanisms and factors involved remain incompletely defined. The present study aimed to investigate whether IL-6 exerts its protective effects via arresting the cell cycle allowing base excision and repair of oxidized DNA after hepatectomy.

**Results:**

Following seventy percent partial hepatectomy (PH) in wild type (WT) mice IL-6 serum levels increased reaching peak levels at 3 hours. This was associated with markers of cell cycle arrest as p21 expression was increased and cyclin D1 and proliferating cell nuclear antigen (PCNA) expression decreased. In the absence of IL-6, markers of cell cycle arrest were absent and the number of bromodeoxyuridine (BrdU) positive cells was significantly higher at 28, 32 and 36 hours after PH. The mRNAs for DNA repair enzymes, including Neil-1, 8-oxodGTPase, OGG1, Apex1, and UDG (DNA glycosylase) were increased 2 to 4 fold in WT mice at 6 and/or 12 hours after PH compared to IL-6 knockout (KO) mice. The protein levels of Neil1 and OGG1 were also significantly increased in WT mice compared to KO mice. Pathological changes were far greater and survival was less in IL-6 KO mice than in WT mice. Administration of IL-6 in KO mice restored p21 and DNA repair enzyme expression to wild-type levels and survival was improved.

**Conclusions:**

IL-6 caused cell cycle arrest and delayed proliferation during the first day after PH. This delay was associated with the activation of DNA repair enzymes resulting in accurate replication and restoration of hepatic mass.

## Background

Liver regeneration in response to partial resection is an efficient and precisely regulated process during which the surviving liver cells proliferate to reconstitute the liver. In rats, the two third partial hepatectomy (~70% PH) induces a compensatory hyperplasia of the remaining lobes that restores the original liver mass within 7 to 10 days [1]. Resection of half of the human liver is followed within 2–4 weeks by full restoration of the liver structure, size, and function [2,3]. However, even in patients without pre-existing liver disease, more significant liver resections, i.e. greater than 75%, result in an increasing perioperative mortality [4].

Interleukin (IL)-6 is a pleiotropic cytokine, which has been shown to provide liver protection in various settings [5-10]. Liver regeneration is impaired in *IL-6* knockout mice [11], while IL-6 overexpression is involved in delayed liver regeneration [12]. While IL-6 has been shown to be vital for liver regeneration [6,13,14], the specific mechanisms and factors involved remain incompletely defined. Identifying the molecular signals that promote regeneration may well lead to therapies that would aid recovery from massive liver injury or surgery.

Over stimulation of TNF-α and increased sensitivity to endotoxin (lipopolysacharides) have been proposed [15] as possible mechanisms for the intracellular production of ROS and lipid peroxidation known to follow 70% PH. While low amounts of ROS may play important roles in liver regeneration after PH, excessive amounts of reactive oxygen species (ROS) are likely to damage cellular DNA. The oxidative injury is more dramatic after large (87%) hepatectomy [14]. Accordingly, we reasoned if the DNA is significantly oxidized and mutated, stringent, robust repair mechanisms would be required to enable injured hepatocytes to proliferate efficiently. We hypothesized that IL-6 exerts its protective effects via arresting the cell cycle allowing base excision and repair of oxidized DNA after hepatectomy.

## Results

### IL-6 Is Important for Survival after 70% PH in Mice

**Figure 1:**
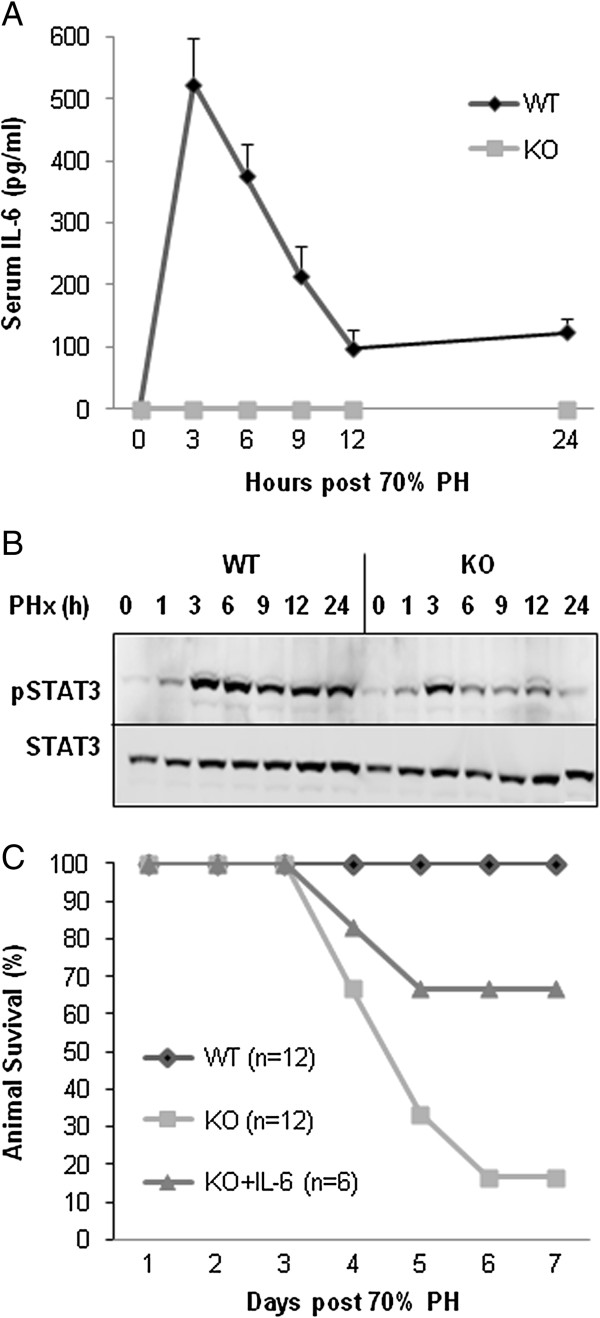
**IL-6 production, Stat3 activation and mortality after PH.** Eight to 10 week-old male IL-6 KO or WT mice were subject to 70% PH and then observed for 7 days or sacrificed at different time points. **(A)** Serum IL-6 levels were measured by ELISA. **(B)** Phosphorylated-Stat3 (p-Stat3) was measured by western blot. P-Stat3 expression was significantly increased in the liver of WT mice compared to IL-6 KO mice. **(C)** Animal survival during the 7 days after 70% PH was 100% for WT mice and 17% for IL-6 KO mice. SC injection of IL-6 in KO mice resulted in survival of 67%.

We performed 70% PH in WT mice, in IL-6 KO mice, and in IL-6 KO mice that were treated 30 minutes before surgery with subcutaneous (SC) injections of recombinant IL-6. Some mice were followed to determine survival rates while others were sacrificed at 0, 3, 6, 9, 12, 24, 32, 48 and 72 hours after partial hepatectomy. The serum level of IL-6 increased dramatically in WT mice, reaching peak levels at 3 hours and remained at a high level at 24 h after PH. No IL-6 was detected in IL-6 KO mice (Figure 1A). IL-6 stimulation was associated with increases in phosphorylated-Stat3 (p-Stat3) in the liver of WT mice compared to IL-6 KO mice (Figure 1B). Animal survival at 7 days after PH was 100% for WT mice and 17% for IL-6 KO mice. SC injection of IL-6 in KO mice resulted in survival of 67% (Figure 1C).

### Increased oxidative DNA damage in IL-6 KO Mice after PH

**Figure 2:**
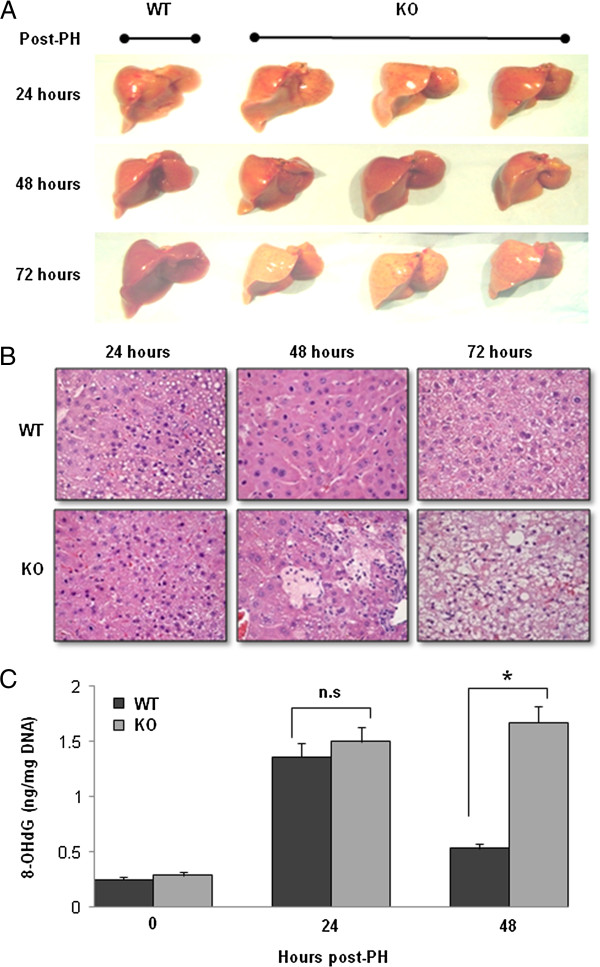
**Oxidative DNA damage in the remaining liver after PH. (A)** The gross morphological changes after 70% PH. There is no difference between WT and IL-6 KO mice on day 1 and day 2 after PH. However, on day 3 after PH, IL-6 KO livers show pale-yellowish color and the liver size did not increase. In contrast, the WT liver increased in size and had a normal color. Representative photographs of n = 3 individual samples per group. **(B)** H&E staining demonstrated that necrosis appeared in liver tissue sections recovered from IL-6 KO mice at 48 and 72 hours after PH in IL-6 KO mice. No necrosis/apoptosis appeared in liver sections from WT mice after PH. **(C)** Levels of 8-OHdG in DNA of the remaining liver after PH. Data represent mean ± SEM (n = 3). n.s., no significant difference. *P < 0.05.

There was no gross morphological difference between WT and IL-6 KO mice on days 1 and 2 (Figure 2A). IL-6 KO livers (middle panel) looked larger than the WT on day 2 after PH. However, on day 3, livers from the mice without IL-6 had a pale-yellowish color and the liver size did not increase further. In contrast, the WT liver increased in size and had a normal color. H and E staining demonstrated that necrosis appeared in liver tissue sections recovered from IL-6 KO mice at 48 hours after PH (Figure 2B), and this progressed to massive areas of necrosis at 72 hours. These features were absent in liver sections from WT mice after PH.

Using a competitive ELISA method, levels of 8-OHdG in total DNA were determined after PH and were found significantly and equally elevated in the DNA of the livers from both WT and IL-6 KO mice at 24 hours (Figure 2C). Interestingly, 8-OHdG levels in the DNA from WT mice decreased significantly at 48 hours after PH, whereas 8-OHdG levels from KO mice remained significantly elevated.

### IL-6 Regulates cell cycle at early time after partial hepatectomy

**Figure 3:**
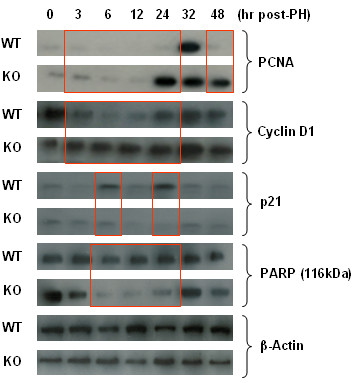
**Expression of cell cycle proteins and PARP after PH.** PCNA, cyclin D1, p21 and PARP in the remaining liver were analyzed by western blot analysis after PH. β-actin was used as loading control. Cyclin-D1 expression was increased as early as 3 hours and remained at the higher levels in IL-6 KO mice after PH. In contrast, there is no increased expression of cyclin-D1 in WT mice until 24 hour after PH. PCNA expression was significantly increased in IL-6 KO mice at 24 hours after PH. However, there is no increased expression of PCNA in WT mice at 24 hours after PH. The expression of cell cycle inhibitor, p21, was significantly higher in WT mice at 6 or 24 hours after HP compared to IL-6 KO mice. Almost no p21 expression was present in IL-6-KO mice at 24 hours after PH. Full length PARP (116 kDa) expression was decreased in IL-6 KO mice compared to WT mice at 6, 12, and 24 hours after PH. Representative data of 3 or 4 individual samples per group.

Using western blot analysis, we found that cyclin-D1 expression was increased as early as 3 hours and remained at the higher levels in the IL-6 KO mice after 70% PH (Figure 3). In contrast, there was no increase of cyclin-D1 in WT mice until 24 hour after 70% PH. Proliferating cell nuclear antigen (PCNA) expression was also significantly increased in IL-6 KO mice at 24 hours after PH while similar to cyclin D-1 there was no increase of PCNA in WT mice. Interestingly, the expression of cell cycle inhibitor, p21, was significantly higher in WT mice at 6 and 24 hours after PH compared to IL-6 KO mice which had minimal if any p21 expression. Figure 3 shows that full length PARP expression was decreased in IL-6 KO mice compared to WT mice at 6, 12, and 24 hours after PH.

**Figure 4:**
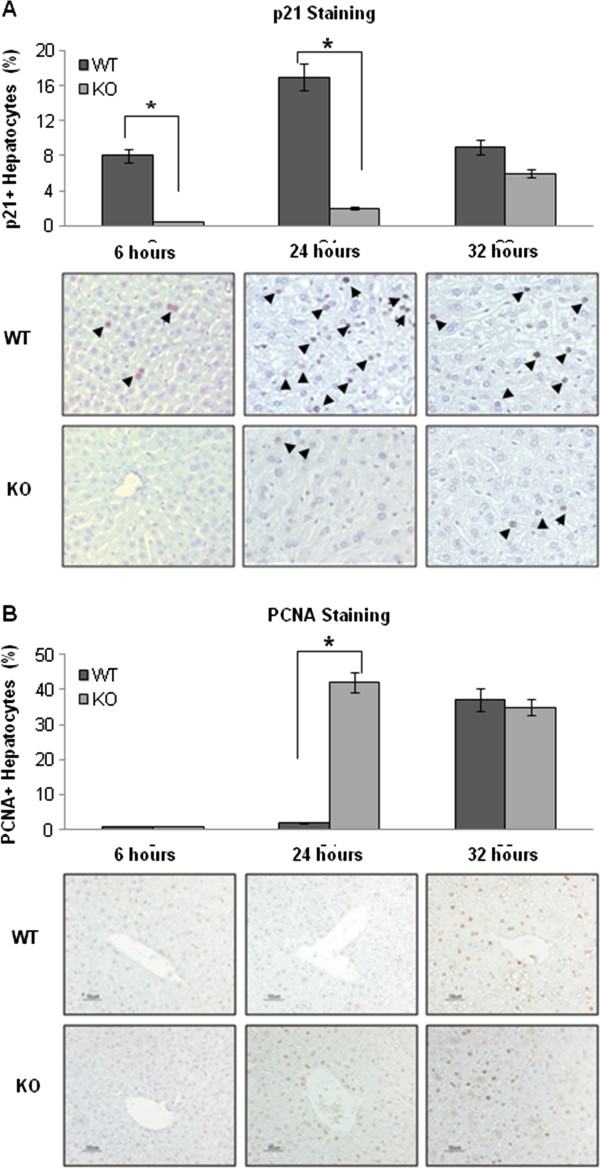
**Immunohistochemistry staining for p21 and PCNA in the remaining liver after PH. (A)** p21 staining. There are more p21 positive hepatocytes in the liver tissue sections from WT mice compared to KO mice at 6 or 24 hours after PH. **(B)** PCNA staining. There were fewer PCNA positive cells in the liver tissue sections from WT mice compared to KO mice at 24 hours after PH. p21 or PCNA positive hepatocytes in each slide were counted in five low-power (×100) fields. Data represent mean ± SEM (n = 3). *P < 0.01.

**Figure 5:**
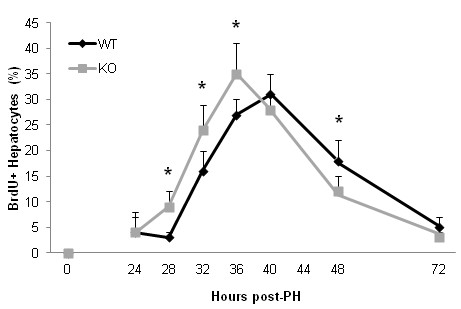
**Hepatocyte proliferation *in vivo *was determined using the bromodeoxyuridine (BrdU) incorporation assay.** Mice were injected (IP) with BrdU (50 μg/g body weight) and euthanized 2 hours later, and the livers were harvested for IHC staining of BrdU using a kit (BD Biosciences). The percentage of BrdU-labeled hepatocytes in each slide was counted in five low-power (×100) fields. Data represent mean ± SEM (n = 3). *P < 0.05.

Immunohistochemistry staining detecting the p21 and PCNA proteins supported the findings above. There were more p21 positive hepatocytes in the liver tissue sections from WT mice compared to KO mice at 6 and 24 hours after PH (Figure 4A). In contrast, there were fewer PCNA positive cells in the liver tissue sections from WT mice compared to KO mice at 24 hours after PH (Figure 4B). The suggestion that acute cell cycle arrest occurred in WT mice was confirmed by BrdU incorporation assays. There was no increase in the number of BrdU positive cells (S phase) in liver tissue sections from WT mice until 32 hours after PH (Figure 5). However, the number of BrdU positive hepatocytes in liver tissue sections from KO mice was significantly increased as early as 28 hours after PH and the number of BrdU positive cells was significant higher in KO mice compared to WT mice at 32 or 36 hours after PH. The data indicate that in the presence of IL-6, replication was stimulated in cells hampered by oxidized DNA.

Activation of DNA repair enzymes occurred in WT mice but not in IL-6 KO mice after 70% PH, and IL-6 pretreatment restored cell cycle arrest and increased DNA repair enzymes.

**Figure 6:**
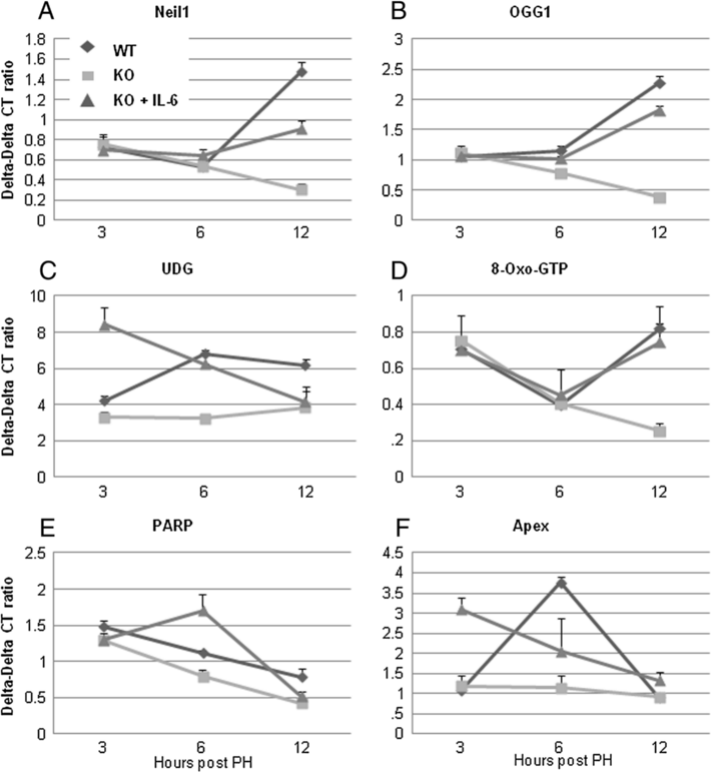
**Real-time PCR analyses for DNA repair enzymes.** The mRNA expression of Neil1, OGG1, 8-oxo-GTP, PARP, UDG and Apex was determined in WT and IL-6 KO mice at 3, 6 and 12 hours after PH using quantitative-real time PCR. Data represent mean ± SEM (n = 3). *P < 0.05 compared to KO mice. **(A)** Neil1, **(B)** OGG1, **(C)** UDG, **(D)** 8-Oxo-GTP, **(E)** PARP, **(F)** Apex.

**Figure 7:**
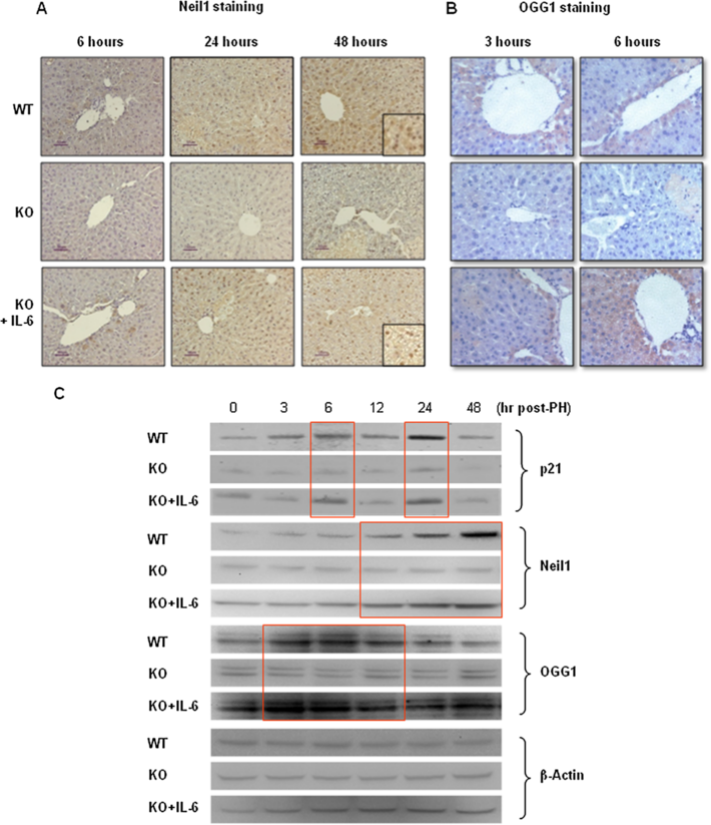
**Detection of Neil1 and OGG1 in the remaining liver after PH. (A)** Immunohistochemical staining for Neil1 **(A)** and OGG1 **(B)**. Positively staining cells appear brown. Representative data of 3 or 4 individual samples per group. The number of Neil1 or OGG1 positive hepatocytes was significantly increased in WT mice or KO mice pretreated with IL-6 compared to KO mice after PH. **(C)** Western blot analysis confirmed the finding that expression of p21, Neil and OGG1 was increased only in WT mice or IL-6 KO mice with SC injection of IL-6. Each lane represents hepatic protein extracted from one animal at each time-point.

We measured the mRNA of several DNA repair enzymes in order to assess whether cell cycle arrest was associated with their upregulation. Using quantitative-real time PCR, the mRNA expression of PARP, UDG and Apex was significantly increased in WT mice compared to KO mice at 6 hours after 70%H, while that of Neil1, OGG1 and 8-oxo-GTP was significantly increased in WT mice compared to KO mice at 12 hours after 70% PH (Figure 6). Neil1 and OGG1 protein levels were also studied by immunohistochemistry and western blot analyses. As shown in Figure 7, the number of Neil1 or OGG1 positive hepatocytes was significantly increased in WT mice or KO mice pretreated with IL-6 compared to KO mice after 7%H (Figure 7A and B). Western blot analysis confirmed the finding that expression of p21, Neil1 and OGG1 was increased only in WT mice or IL-6 KO mice with SC injection of IL-6 (Figure 7C).

## Discussion

The expected burst of IL-6 secretion was present after 70% PH, but instead of finding robust measures of early proliferation we found depressed BrdU incorporation and evidence of cell cycle arrest as p21 expression was increased and cyclin-D1 and PCNA expression was depressed. IL-6 KO mice had the reverse pattern with greater early incorporation of BrdU, absent p21 and increased cyclin-D1 and PCNA levels. To explain this seeming paradox where normal mice had evidence of cellular slow down and the IL-6 deficient mice were geared to replicate, we proposed that cell cycle arrest was needed to create a check point to allow for activation of DNA repair enzymes and to have them excise and replace oxidized DNA thereby permitting accurate replication.

Using measurements of the stable oxidized adduct 8-OHdG as an indication of DNA oxidative injury, we confirmed that significant oxidative injury occurred equally in both IL-6 KO and WT mice at 24 hours after PH. However, by day two there was little 8-OHdG in the WT mice and much more in the IL-6 KO mice. This was associated with striking differences in histology as the WT mice displayed normal histology while the IL-6 KO mice had extensive areas of necrosis. The best explanation for this difference is that the absence of a check point in the IL-6 KO mice resulted in rapid but faulty DNA replication leading to apoptosis/necrosis.

The mechanism leading to cell cycle arrest is likely related to the increased expression of p21 found in the WT mice at 6 and 24 hours after 70% PH compared to IL-6 KO mice. We have shown in previous studies [16-19] that IL-6 significantly increased phosphorylated STAT3, which stimulated p21 transcription [20-23] resulting in G-1 arrest. We propose that normal mice had sufficiently high levels of IL-6 after 70% PH to increase p21 production via activated STAT3 thereby leading to cyclin D-1 and PCNA expression and to G-1/S arrest. This hypothesis is supported by the finding that rIL-6 stabilized steatotic hepatocytes from obese animals by normalizing PCNA expression (G1 phase) and not by increasing DNA synthesis (BrdU, S phase) [24].

We have found that the expression of p21 was significantly higher in WT mice at 6 and 24 hours, but not 12 hours after PH compared to IL-6 KO mice which had minimal if any p21 expression. Interestingly, the expression of p21 was increased only at 6 and 24 hours in IL-6 KO mice with SC injection of IL-6. It has been reported that two waves of STAT3 activation were observed after PH, the first in endothelia and the second in hepatocytes, and STAT3 activation was positively correlated with p21 protein expression [25]. Two peaks of p21 expression at early time after PH might be due to IL-6 dependent activation of STAT3.

Direct evidence supporting the key role of DNA repair comes from the finding that the principle known enzymes for DNA repair, OGG-1, 8-oxo-GTP, Neil-1 were stimulated in the WT mice but absent in the IL-6 KO mice following 70% PH. PARP which repairs single strand breaks most commonly caused by per oxidation was also found increased at early time points in WT mice. The crucial role of IL-6 is supported by the restoration of early cell cycle arrest in IL-6 KO mice and subsequent benefit by a single injection 30 minutes before 70% PH.

Other studies have also linked IL-6 with DNA repair. Efferth et al. [26] reported that IL-6 improved DNA repair in melphalan-induced DNA damaged human multiple myeloma cells. Other reports have shown that IL-6 mRNA is stimulated after DNA strand breaks [27] and persistent DNA damage triggers senescence-associated inflammatory cytokine secretion including IL-6 [28,29]. We have shown that activation of DNA repair enzymes occurred in WT mice and could be restored in IL-6 KO mice by IL-6 injection just before 70% PH.

In conclusion, we provide evidence that the essential role of IL-6 in liver regeneration after 70% PH is the induction of cell cycle checkpoints and activation of DNA repair enzymes in the remaining liver. IL-6 promoted DNA repair, but was not a requirement for liver cell proliferation. Cell cycle arrest permitted time for the transcription of DNA repair enzymes, DNA base excision repair and subsequent accurate cellular replication.

## Materials and methods

### Animals and partial hepatectomy

Male C57BL/6 J background IL-6 KO mice and control C57BL/6 J (WT) mice weighing 20–25 g were purchased from the Jackson Laboratory (Bar Harbor, ME). Animals were maintained in a pathogen-free facility of The Johns Hopkins Medical Institutions. Animals were cared for according to NIH guidelines and under a protocol approved by the Johns Hopkins University Animal Care Committee. For the IL-6-injected group, IL-6 KO mice were given a subcutaneous injection with recombinant IL-6 at a dose of 500 ng/g of animal weight thirty minutes before hepatectomy. Animals were anesthetized with isofluorane. After a midline laparotomy, the left and middle lobes (~70%) of the liver were ligated at the base and removed. The abdominal wall and skin were sutured separately. After surgery, animals were immediately given free access to water. Following surgery, mice were sacrificed, and livers and caval blood samples were collected at predetermined time points after operation. Harvested livers were weighted to assess regeneration, and portions of liver tissue were either fixed in 10% neutralized formalin for histological evaluation or were snap frozen in liquid nitrogen and maintained at −80°C until homogenization for various biochemical assays. For assessment of hepatic proliferation, BrdU was injected (50 mg/kg intraperitoneal (IP)) 2 hours prior to harvesting of liver.

### Analysis of alanine aminotransaminase and aspartate aminotransaminase activity

Acute liver injury was quantified by measurement of serum alanine aminotransaminase (ALT) and aspartate aminotransaminase (AST) levels using an automated enzyme assay.

#### Histopathological analysis

Cut sections of 4 μm were prepared from formalin-fixed paraffin-embedded tissues for immunohistochemistry. Each representative section was stained with hematoxylin-eosin (H&E). Immunohistochemical staining was performed with the avidin-biotin-peroxidase complex method, using Vectastain ABC kits (Vector Laboratories, Burlingame, CA) according to the methods described before [19,28,30]. The following antibodies were used: mouse anti-BrdU 1monoclonal antibody (Molecular Probes) (1:200); anti-PCNA antibody (Cell Signaling) (1: 5000); polyclonal anti-OGG1 antibody (1:100) (Novus Biological, Littleton, CO); monoclonal anti-p21 antibody (1:200) and polyclonal anti-Neil1 antibody (1:100) (Cell Signaling Technology, Boston, MA). The antibodies were incubated either at 4°C overnight or at room temperature for 1.5 hours. Immunohistochemistry staining of bromodeoxyuridine (BrdU) was performed by using a BrdU incorporation assay kit (BD Biosciences).

### ELISA assay for measurement of 8-OHdG Levels in DNA

The DNA was extracted using the DNeasy tissue kit (Qiagen, Santa Clara, CA) according to the manufacturer’s instruction [28]. The levels of 8-OHdG in nucleoside samples were used for the determination of 8-OHdG by a competitive ELISA kit (8-OHdG Check, Institute for the Control of Aging). The determination range was 0.125–10 ng/ml or 0.5–200 ng/ml. The levels of 8-OHdG were expressed as amounts of 8-OHdG (ng) per milligram of DNA.

### Western blot analysis

Tissues were homogenized in lysis buffer (30 mmol/L Tris, pH 7.5, 150 mmol/L sodium chloride, 1 mmol/L phenylmethylsulfonyl fluoride, 1 mmol/L sodium orthovanadate, 1% Nonidet P-40, 10% glycerol) at 4°C, vortexed and centrifuged at 16,000 rpm at 4°C for 10 minutes. The supernatants were mixed in SDS sample loading buffer (NuPAGE, Invitrogen), 70°C for 10 minutes, and then subjected to SDS-PAGE. After electrophoresis, proteins were transferred onto PVDF membranes and blotted against primary antibodies (anti-p21 (1:2000), anti-Neil1 (1:1000), anti-OGG1 (1:1000), anti-PARP (1:1000, #9542, Cell Signaling Technology) and anti-β-actin (1:1000)) for 1 hour at room temperature. Membranes were washed with TPBS (0.05% [vol/vol] Tween 20 in phosphate-buffered saline [pH 7.4]) and incubated with a 1:4000 dilution of horseradish peroxidase-conjugated secondary antibodies for 45 minutes. Protein bands were visualized by an enhanced chemiluminescence reaction (ECL-plus, Bio-Rad).

### Real-Time PCR Analysis for DNA repair enzyme mRNA

The primer sets for amplification of mouse Neil1 were 5’-ACCCTGTGTCTTGCTGGAGT-3’ and 5’-TGCAGGGCCTCTAGAACTGT-3’. The primer sets for amplification of mouse OGG1 were 5’-GACTACGGCTGGCATCCTAAG-3’ and 5’-GCAAAAAGGGATCTAAGAGGC-3’. The primer sets for control amplification of glyceraldehyde 3-phosphate dehydrogenase (GAPDH) were 5’-ATTCAACGGCACAGTCAAGG-3’ and 5’- CACACCCATCACAAACATGG-3’. For the Real-Time PCR procedure: The cDNA was diluted 1:10 and 2 μl were used as template in a 25 μl qPCR reaction. The qPCR assays were performed as described previously, using SYBR Green (Applied Biosystems, Foster City, CA) on the IQ5(Bio-Rad). Amplification conditions were 95°C for 5 minutes and cycles of 95°C for 30 seconds, 59°C for 30 seconds, and 72°C for 30 minute, with a final extension at 72°C for 5 minutes. All PCR reactions were performed in triplicate. After amplification, we determined the threshold cycle (Ct) to obtain expression values of 2^–Ct^, as described previously. A ΔCT value was first calculated by subtracting the CT value for the housekeeping gene GAPDH from the CT value for each sample. A ΔΔCT value was then calculated by subtracting the ΔCT value of the control (normal control) from the ΔCT value of each treatment.

### Statistics

The results were expressed as mean values ± SEM of n independent experiments. Analyses were performed using the paired Student *t* test and the analysis of variance (ANOVA) with repeated measures. P values less than .05 was considered a significant difference between KO mice and WT mice.

## Abbreviations

Apex: DNA-(apurinic or apyrimidinic site) lyase; BrdU: Bromodeoxyuridine; IL-6: Interleukin-6; KO: Knock out; Neil1: Nei endonuclease VIII-like 1; OGG1: 8-Oxoguanine glycosylase; 8-OHdG: 8-hydroxy-2' –deoxyguanosine; 8-oxo-GTP: 8-oxoguanine derivative of GTP; PARP: Poly ADP ribose polymerase; PCNA: Proliferating cell nuclear antigen; PH: Partial hepatectomy; p-Stat3: phosphorylated-Stat3; p21: CDK-interacting protein 1; ROS: Reactive oxygen species; SC: Subcutaneous; UDG: Uracil-DNA glycosylase; WT: Wild type.

## Competing interests

The authors declare that they have no competing interests. The authors alone are responsible for the content and writing of the paper.

## Authors’ contributions

ST, XZ and KI performed majority of the experiments, analyzed data and prepared the manuscript. Specifically, ST and KI performed PH and western blot analysis, XZ performed histological analysis, ELISA and PCR. AMC and GMW provided suggestions for the project and edited the manuscript. ZS designed the overall study, supervised the project, and wrote the manuscript. All authors read and approved the final manuscript.
